# Correlation analysis between disease severity and clinical and biochemical characteristics of 143 cases of COVID-19 in Wuhan, China: a descriptive study

**DOI:** 10.1186/s12879-020-05242-w

**Published:** 2020-07-16

**Authors:** Dan Wang, Ruifang Li, Juan Wang, Qunqun Jiang, Chang Gao, Juan Yang, Lintong Ge, Qing Hu

**Affiliations:** 1 Department of Neurology, Hubei NO.3 People’s Hospital of Jianghan University, 26 Zhongshan Road, Qiaokou District, Wuhan, China; 2 Department of Radiology, Tongji Hospital, Huazhong University of Science and Technology, Wuhan, China; 3 Department of Infectious Diseases, Zhongnan Hospital of Wuhan University, Wuhan, China

**Keywords:** SARS-CoV-2, COVID-19, Correlation analysis, Prognostic indicator

## Abstract

**Background:**

Coronavirus disease 2019 (COVID-19) is a novel infectious disease caused by the severe acute respiratory syndrome coronavirus 2 (SARS-CoV-2) emerged in Wuhan and has quickly spread across the world. The mortality rate in critically ill patients with COVID-19 is high. This study analyzed clinical and biochemical parameters between mild and severe patients, helping to identify severe or critical patients early.

**Methods:**

In this single center, cross-sectional study, 143 patients were included and divided to mild/moderate and sever/critical groups. Correlation between the disease criticality and clinical features and peripheral blood biochemical markers was analyzed. Cut-off values for critically ill patients were speculated through the ROC curve.

**Results:**

Significantly, disease severity was associated with age (*r* = 0.458, *P* < 0.001), comorbidities (*r* = 0.445, *P* < 0.001), white cell count (*r* = 0.229, *P* = 0.006), neutrophil count (*r* = 0.238, *P* = 0.004), lymphocyte count (*r* = − 0.295, *P* < 0.001), albumin (*r* = − 0.603, *P* < 0.001), high-density lipoprotein cholesterol (*r* = − 0.362, *P* < 0.001), serum potassium (*r* = − 0.237, *P* = 0.004), plasma glucose (*r* = 0.383, *P* < 0.001), total bilirubin (*r* = 0.340, *P* < 0.001), serum amyloid A (*r* = 0.58, *P* < 0.001), procalcitonin (*r* = 0.345, *P* < 0.001), C-reactive protein (*r* = 0.477, *P* < 0.001), lactate dehydrogenase (*r* = 0.548, *P* < 0.001), aspartate aminotransferase (*r* = 0.342, *P* < 0.001), alanine aminotransferase (*r* = 0.264, *P* = 0.001), erythrocyte sedimentation rate (*r* = 0.284, *P* = 0.001) and D-dimer (*r* = 0.477, *P* < 0.001) .

**Conclusions:**

With the following parameters such as age > 52 years, C-reactive protein > 64.79 mg/L, lactate dehydrogenase > 245 U/L, D-dimer > 0.96 μg/mL, serum amyloid A > 100.02 mg/L, or albumin < 36 g/L, the progress of COVID-19 to critical stage should be closely observed and possibly prevented. Lymphocyte count, serum potassium, high-density lipoprotein cholesterol and procalcitonin may also be a prognostic indicator.

## Background

A novel coronavirus, designated as the severe acute respiratory syndrome coronavirus 2 (SARS-CoV-2), was first identified in Wuhan, China in December 2019 [1]. SARS-CoV-2 is highly infectious and asymptomatic patients may also become the source of infection [2]. World Health Organization (WHO) announced that the disease caused by SARS-CoV-2 was coronavirus disease 2019 (COVID-19) on February 11, 2020. Patients with COVID-19 have a series of clinical manifestations, such as pharyngalgia, fever, cough, fatigue, anorexia, headache, diarrhea, nausea or vomiting, dyspnea [3], even acute respiratory distress syndrome (ARDS). A lot of severe or critical patients had to been admitted to the intensive care unit (ICU). According to the reported clinical characteristics of patients with COVID-19, the total mortality ranges from 2 to 5%, which can be even higher in the elders [4]. Wuhan city, as the epidemic area, the mortality reached a peak of over 7% at the early stage [5].

Although most patients with COVID-19 were mild in the early days, some patients progressed rapidly to acute respiratory failure, metabolic acidosis, septic shock, ARDS or death. Early identification of risk factors for critical patients could facilitate appropriate supportive care and thus reduce the mortality [6]. A study of the first 138 laboratory-confirmed cases with COVID-19 showed the changes of neutrophil count, lymphocyte count, and D-dimer levels [7]. Increased inflammation -related indicators were found in patients with COVID-19, including erythrocyte sedimentation rate (ESR), interleukin-6 and C-reactive protein (CRP) [4]. However, little is known about the relationship between disease severity and clinical and biochemical features in patients with COVID-19.

In this study, we performed a comprehensive evaluation of characteristics of 143 patients with COVID-19 admitted to Hubei NO.3 People’s Hospital of Jianghan University, Wuhan. This study retrospectively analyzed clinical characteristics and biochemical parameters between mild/moderate and severe/critical patients, which may help to identify critical cases and perform appropriate clinical intervention early.

## Methods

### Study design and participants

This study was a cross-sectional study, and all consecutive patients with confirmed COVID-19 admitted to Hubei NO.3 People’s Hospital of Jianghan University from January 15, 2020 to February 28, 2020, were enrolled. Hubei NO.3 People’s Hospital of Jianghan University, located in Wuhan, Hubei Province, the endemic areas of SARS-CoV-2, is one of the major public hospitals and is responsible for the treatments for COVID-19 assigned by the government. Diagnosis of COVID-19 and clinical classification according to the new coronavirus pneumonia diagnosis and treatment plan (trial version 7) developed by the National Health Commission of the People’s Republic of China [8].

The clinical classifications are as follows: (1) mild, minor symptoms and imaging shows no pneumonia. (2) moderate, with fever, respiratory tract symptoms, and imaging shows pneumonia. (3) severe, meet any of the following: a) respiratory distress, respiratory rate ≥ 30 beats/min; b) in the resting state, means oxygen saturation ≤ 93%; c) arterial blood oxygen partial pressure/oxygen concentration ≤ 300 mmHg (1 mmHg = 0.133 kPa); d) pulmonary imaging showed that the lesion progressed more than 50% within 24–48 h. (4) critical, one of the following conditions: a) respiratory failure occurs and requires mechanical ventilation; b) Shock occurs; c) ICU admission is required for combined organ failure.

In this study, the patients with mild or moderate symptoms were classified as mild/moderate group, and the patients with severe or critical symptoms were classified as severe/critical group. The assessment of disease severity and laboratory tests were performed at the same time on the day of inpatient admission before treatment.

### Data collection

All suspected infection patients were taken upper respiratory throat swab samples at admission and then shipped to designated authoritative laboratories to detect the SARS-CoV-2. Bacterial and fungal detections of sputum or respiratory secretions and other laboratory tests were completed in the clinical laboratory in Hubei NO.3 People’s Hospital of Jianghan University. C-reactive protein (CRP) was detected by immunoturbidimetry method. Procalcitonin (PCT) was detected by Roche electrochemiluminescence method. Erythrocyte sedimentation rate (ESR) was measured by Westergren’s international standard method.

We retrospectively analyzed and evaluated the epidemiological history, comorbidities, vital signs, and symptoms obtained from electronic medical records. The data collection forms were reviewed independently by two experienced physicians. These patients have not been reported in any other submission by anyone.

### Statistical analysis

Categorical variables were given as frequency rates and percentages; continuous variables were defined using mean, median, and interquartile range (IQR) values. The Kolmogorov-Smirnov test was used to verify the normality of distribution of continuous variables. The independent sample t test or the Mann-Whitney U test was used for the continuous variables and the chi-square test for the categorical variables. In correlation analysis, Pearson correlation coefficient was used for the variables of normal distribution and Spearman correlation coefficient for those of skewed distribution. Receiver–operating characteristic (ROC) curve analysis was used to determine the optimum cut-off points of parameters for severe patients. Statistical analyses were performed using SPSS 24.0 (SPSS Inc., Chicago, IL, USA) and MedCalc 19.0.4. A 2-tailed *P* < 0.05 was considered as statistically significant.

## Results

**Fig. 1:**
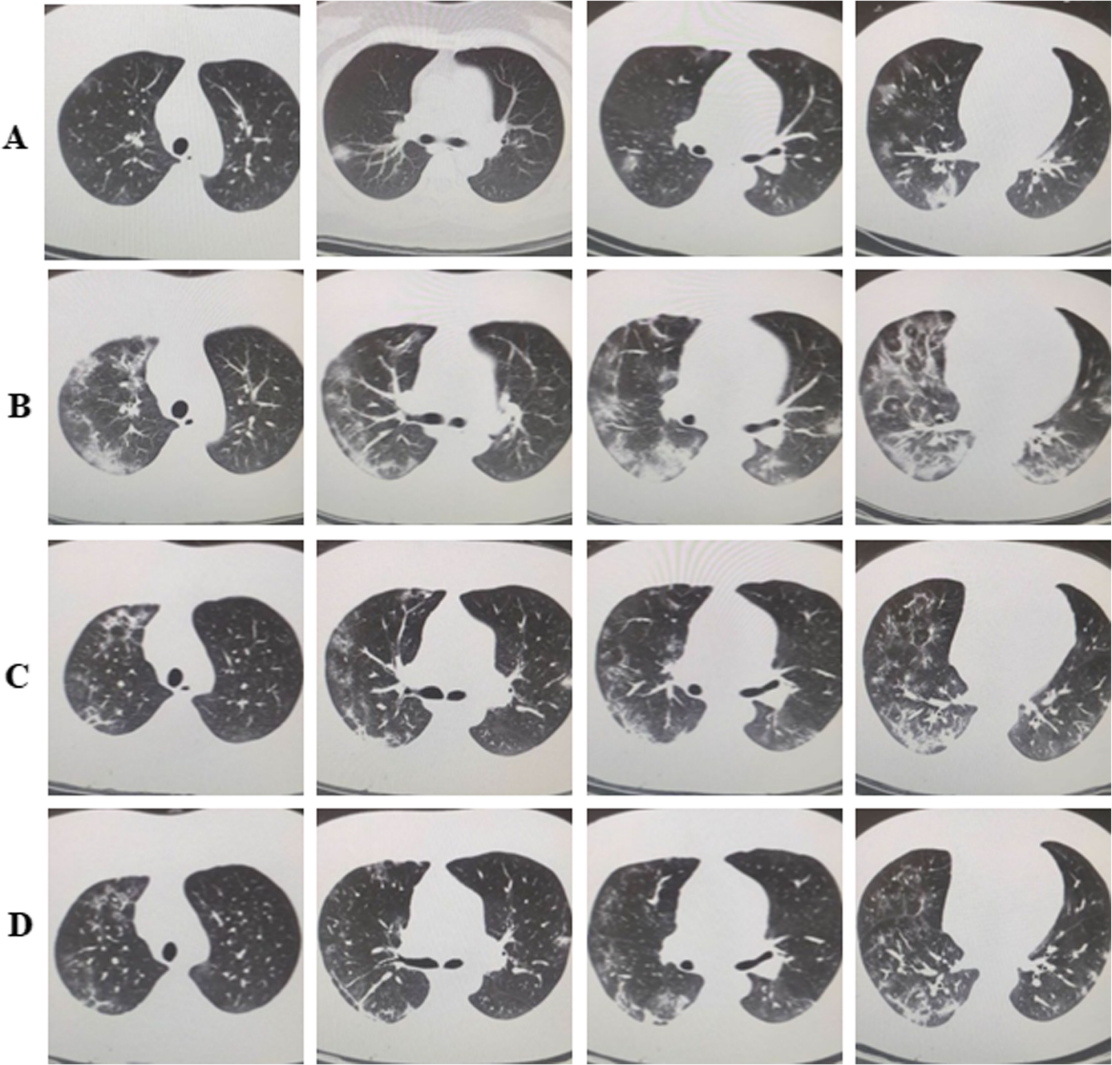
A patient with severe acute respiratory syndrome coronavirus 2 (SARS-CoV-2) infection. **a** Ground glass shadow in multiple lobes and segments of bilateral lungs, and the lesions were adjacent to the pleura (Illness Day 7, Hospital Day 0). **b** Ground glass shadow expanding and consolidation in bilateral lung (Illness Day 12, Hospital Day 5). **c** Ground glass shadow absorption and reduced consolidation area (Illness Day 18, Hospital Day 11). **d** Lesion dissipation (Illness Day 24, Hospital Day 17)

The study population included 143 hospitalized patients with confirmed COVID-19. The median age was 58 years (IQR, 39–67; range, 14–84 years), and 73 (51.0%) were men. Of the 143 patients, 50 (35.0%) had 1 or more comorbidities. Hypertension (36 [25.2%]), cardiovascular disease (16 [11.2%]) and diabetes (13 [9.1%]) were the most common coexisting conditions. The most common symptoms at initial stage of illness were fever (137 [95.8%]), fatigue (93 [65.0%]), dry cough (78 [54.5%]), anorexia (66 [46.2%]), chest tightness (63 [44.1%]), myalgia (49 [34.3%]), mild shortness of breath (48 [33.6%]), chill (33 [23.1%]) and dyspnea (31 [21.7%]). Less common symptoms were nausea or vomiting, diarrhea and headache (Table 1). X-ray or CT showed multiple lung lobes or bilateral involvement in 138 (96.5%) patients. Figure 1 showed the CT images of a typical patient in early, consolidation, absorption and dissipation stages.

**Table 1:** Demographics and baseline characteristics of patients with COVID-19

	No. (%)			***P*** Value ^**a**^
	Total(***N*** = 143)	Mild/Moderate (***n*** = 72)	Severe/Critical(n = 71)	
Age, median (IQR), y	58 (39–67)	44 (32–60)	65 (53–69)	0.000
Sex				0.009
Female	70 (49.0)	43 (59.7)	27 (38.0)	
Male	73 (51.0)	29 (40.3)	44 (62.0)	
Huanan Seafood Wholesale Market exposure	11 (7.7)	4 (5.6)	7 (9.7)	0.359
Comorbidities	50 (35.0)	10 (13.9)	40 (56.3)	0.000
Hypertension	36 (25.2)	5 (6.9)	31 (43.7)	0.000
Cardiovascular disease	16 (11.2)	4 (5.6)	12 (16.9)	0.031
Diabetes	13 (9.1)	4 (5.6)	9 (12.7)	0.139
Chronic obstructive pulmonary disease	10 (7.0)	3 (4.2)	7 (9.9)	0.091
Cerebrovascular disease	5 (3.5)	2 (2.8)	3 (4.2)	0.987
Signs and symptoms				
Fever	137 (95.8)	70 (97.2)	67 (94.4)	0.619
Fatigue	93 (65.0)	42 (58.3)	51 (71.8)	0.091
Dry cough	78 (54.5)	37 (51.4)	41 (57.7)	0.455
Anorexia	66 (46.2)	23 (31.9)	43 (60.6)	0.001
Chest tightness	63 (44.1)	29 (40.3)	34 (47.9)	0.359
Myalgia	49 (34.3)	18 (25.0)	31 (43.7)	0.019
Mild shortness of breath	48 (33.6)	33 (45.8)	15 (21.1)	0.002
Chill	33 (23.1)	19 (26.4)	14 (19.7)	0.344
Dyspnea	31 (21.7)	10 (13.9)	21 (29.6)	0.023
Pharyngalgia	28 (19.6)	12 (16.7)	16 (22.5)	0.377
Diarrhea	26 (18.2)	12 (16.7)	14 (19.7)	0.636
Expectoration	22 (15.4)	5 (6.9)	17 (23.9)	0.005
Nausea or Vomiting	14 (9.8)	7 (9.7)	7 (9.9)	0.978
Headache	7 (4.9)	3 (4.2)	4 (5.6)	0.985
Multiple lung lobe or bilateral involvement	138 (96.5)	68 (94.4)	70 (98.6)	0.371
Onset of symptom to Hospital admission, median (IQR), d	5 (3–7)	4 (3–5)	6 (5–7)	0.000

*Abbreviations: IQR* interquartile range, *COVID-19* Coronavirus disease 2019

^a^*P* values indicate differences between mild/moderate and severe/critical. *P* < 0.05 was considered statistically significant

Compared with the mild/moderate group (*n* = 72), the severe/critical group (*n* = 71) were significantly older (median age, 65 years [IQR, 53–69] vs 44 years [IQR, 32–60]; *P* < 0.001) and were more likely to have underlying comorbidities, including hypertension (31 [43.7%] vs 5 [6.9%], and cardiovascular disease (12 [16.9%] vs 4 [5.6%]). Compared with the mild/moderate group, the severe/critical group were more likely to report anorexia, expectoration, mild shortness of breath, dyspnea and myalgia.

There were numerous differences in laboratory findings between mild/moderate group and severe/critical group, including white blood cell, neutrophil count, and lymphocyte count, as well as levels of high-density lipoprotein cholesterol (HDL-C), plasma glucose, serum potassium, total bilirubin (TBIL), albumin, aspartate aminotransferase (AST), alanine aminotransferase (ALT), lactate dehydrogenase (LDH), D-dimer, erythrocyte sedimentation rate (ESR), serum amyloid A (SAA), C-reactive protein (CRP) and procalcitonin (PCT) (Table 2).

**Table 2:** Laboratory findings of patients with COVID-19 on admission to hospital

	Normal Range	Median (IQR)			***P*** Value^**a**^
		Total (***N*** = 143)	Mild/Moderate (***n*** = 72)	Severe/Critical (***n*** = 71)	
White blood cell count, × 10^9^/L	3.5–9.5	3.8 (3.2–5.8)	3.6 (3.2–4.4)	4.6 (3.2–7.3)	0.006
Neutrophil count, ×10^9^/L	1.8–6.3	3.7 (2.2–5.6)	3.2 (2.2–4.5)	4.5 (2.4–6.6)	0.005
Lymphocyte count, ×109/L	1.1–3.2	0.9 (0.6–1.2)	1.1 (0.7–1.3)	0.7 (0.6–1.0)	0.000
Monocyte count, × 109/L	0.1–0.6	0.3 (0.2–0.4)	0.3 (0.2–0.4)	0.3 (0.2–0.4)	0.816
Hemoglobin, g/L	115–150	123 (119–133)	124 (119–136)	123 (116–130)	0.118
Platelet count, ×109/L	125–350	155 (128–230)	156 (128–228)	154 (124–236)	0.990
HDL-C, mmol/L	1.29–1.55	0.9 (0.8–1.2)	1.1 (0.9–1.3)	0.9 (0.7–1.0)	0.000
LDL-C, mmol/L	< 3.12	2.6 (2.2–3.0)	2.6 (2.1–3.0)	2.7 (2.2–3.0)	0.615
Plasma glucose, mmol/L	3.89–5.83	6.7 (5.7–8.2)	5.8 (5.2–7.5)	7.4 (6.1–9.1)	0.000
Serum sodium, mmol/L	135–145	137.2 (135–140)	138 (136–140)	137 (134–140)	0.119
Serum potassium, mmol/L	3.5–5.5	3.4 (3.2–3.6)	3.5 (3.3–3.6)	3.3 (3.1–3.6)	0.005
Creatinine, μmol/L	44–120	65 (52–76)	61 (51–72)	6 (52–79)	0.175
BUN, mmol/L	2.5–6.7	3.6 (2.7–5.2)	3.5 (2.7–4.8)	4.02 (2.7–5.2)	0.225
TBIL, mmol/L	3.4–20.5	14.4 (10.3–20.0)	13.5 (8.0–16.4)	16.2 (13.4–21.8)	0.000
Albumin, g/L	34–54	36.0 (31.7–39.4)	39.0 (36.9–40.0)	32.0 (30.2–34.0)	0.000
AST, U/L	8–40	51.0 (34.1–72.6)	42.6 (30.3–56.8)	61.0 (43.5–87.0)	0.000
ALT, U/L	5–35	40.0 (22.0–62.4)	35.8 (18.3–46.7)	50.0 (28.8–76.8)	0.002
LDH, U/L	109–245	256 (178–354)	214 (153–257)	321 (256–471)	0.000
CK-MB, U/L	0–25	12.3 (9.8–19.1)	11.5 (9.8–16.7)	12.8 (9.9–20.6)	0.134
D-dimer, ug/mL	0–1	0.5 (0.4–1.5)	0.4 (0.3–0.7)	1.2 (0.5–2.9)	0.000
APTT, s	20–40	27.4 (24–31.5)	26.2 (23.8–31.5)	27.6 (24.2–30.7)	0.477
PT, s	9–14	11.2 (10.8–12.4)	11.1 (10.5–12.3)	11.4 (10.8–12.9)	0.347
ESR, mm/h	0–20	37.4 (18.1–62.4)	25.4 (16.7–48.6)	44.7 (21.4–81.0)	0.001
SAA, mg/L	0.1–10	185.0 (26.1–638.9)	40.6 (13.6–141.0)	477.7 (209–996)	0.000
CRP, mg/L	0–5	15.3 (5.0–69.1)	8.6 (4.7–28.6)	54.8 (11.5–100.5)	0.000
PCT, ng/mL	< 0.04				0.000
< 0.04, n (%)		85 (59.4)	55 (76.4)	30 (42.3)	
0.04–0.25, n (%)		39 (27.3)	12 (16.7)	27 (38.0)	
0.25–0.5, n (%)		14 (9.8)	4 (5.6)	10 (14.1)	
≥0.5, n (%)		5 (3.5)	1 (1.4)	4 (5.6)	

*Abbreviations: HDL-C* high-density lipoprotein cholesterol, *LDL-C* low-density lipoprotein cholesterol, *BUN* blood urea nitrogen, *TBIL* total bilirubin, *AST* aspartate aminotransferase, *ALT* alanine aminotransferase, *LDH* lactate dehydrogenase, *CK-MB* creatine kinase-muscle and brain type, *APTT* activated partial thromboplastin time, *PT* prothrombin time, *ESR* erythrocyte sedimentation rate, *SAA* serum amyloid A, *CRP* C-reactive protein, *PCT* procalcitonin, *IQR* interquartile range, *COVID-19* Coronavirus disease 2019

^a^*P* values indicate differences between mild/moderate and severe/critical. *P* < 0.05 was considered statistically significant

Significant correlations were found about age, comorbidities, white blood cell count, neutrophil count, lymphocyte count, plasma glucose, serum potassium, albumin, D-dimer, HDL-C, TBIL, AST, ALT, LDH, ESR, SAA, CRP and PCT. Strikingly, this analysis revealed negative correlation between disease severity and lymphocyte count, albumin, serum potassium, and HDL-C (Table 3). Age (r = 0.458), comorbidities (r = 0.445), LDH (r = 0.548), D-dimer (r = 0.477), SAA (r = 0.58), CRP (r = 0.477) were moderately correlated and albumin (r = − 0.603) was highly correlated.

**Table 3:** Correlation coefficient and *P* value between items and disease severity

	r	P
Age	0.458	0.000
Comorbidities	0.445	0.000
White blood cell count	0.229	0.006
Neutrophil count	0.238	0.004
Lymphocyte count	−0.295	0.000
HDL-C	−0.362	0.000
Plasma glucose	0.383	0.000
Serum potassium	− 0.237	0.004
TBIL	0.340	0.000
Albumin	−0.603	0.000
AST	0.342	0.000
ALT	0.264	0.001
LDH	0.548	0.000
D-dimer	0.477	0.000
ESR	0.284	0.001
SAA	0.58	0.000
CRP	0.477	0.000
PCT	0.345	0.000

*Abbreviations: HDL-C*, high-density lipoprotein cholesterol, *TBIL* total bilirubin, *AST* aspartate aminotransferase, *ALT* alanine aminotransferase, *LDH* lactate dehydrogenase, *ESR* erythrocyte sedimentation rate, *SAA* serum amyloid A, *CRP* c-reactive protein, *PCT* procalcitonin

**Fig. 2:**
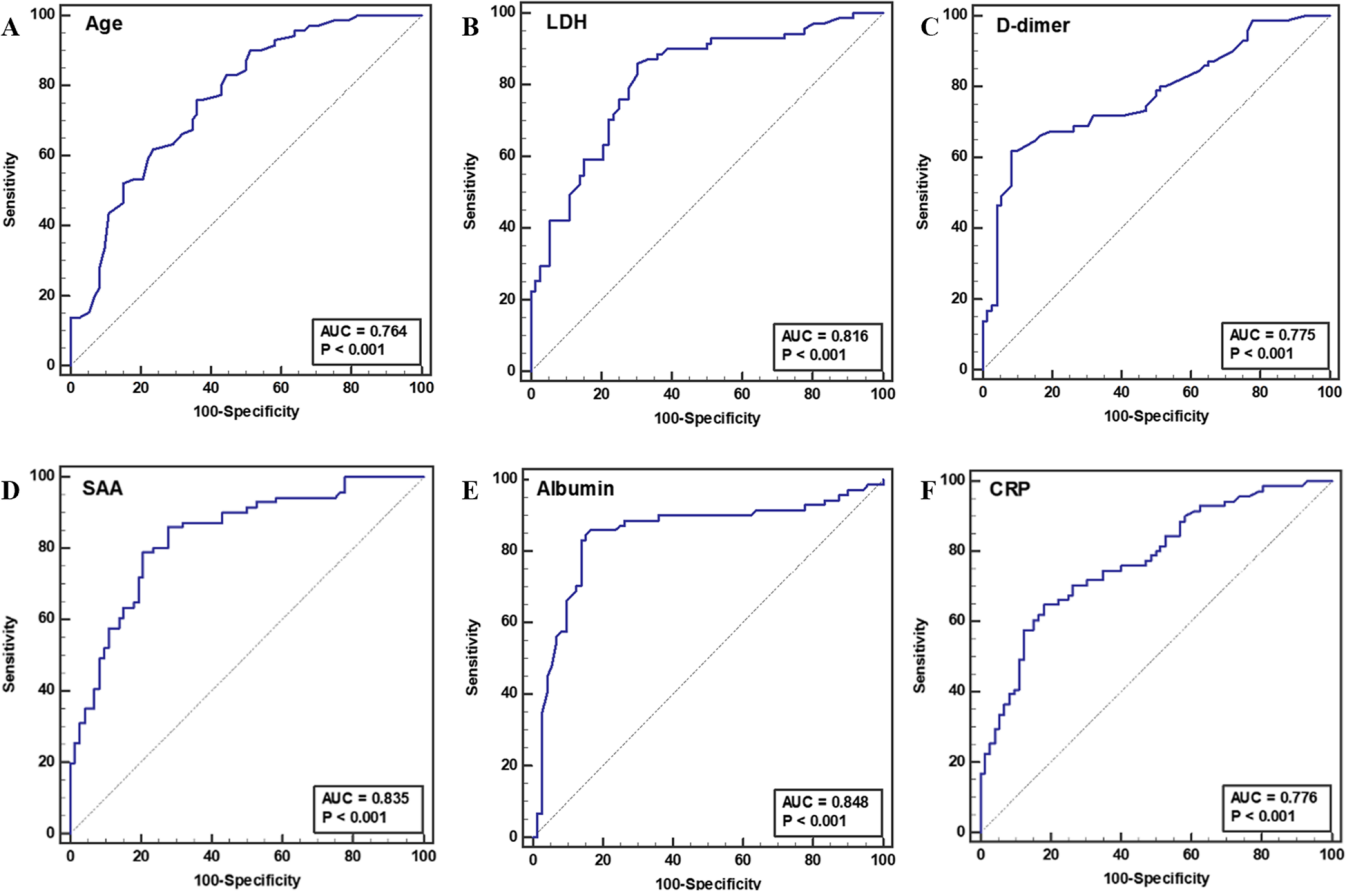
ROC curve of indicators between mild/moderate and severe/critical. **a** age; **b** LDH; **c** D-dimer; **d** SAA; **e** Albumin; **f** CRP

To better detect the severe illness, the ROC curve of age was administrated and listed in Fig. 2a (AUC = 0.746, 95% CI: 0.686–0.831, *P* < 0.001). The best cut-off point of age was 52 years with a sensitivity of 76.1% and specificity of 63.9%. The ROC curve of LDH (AUC = 0.816, 95% CI: 0.743–0.876, *P* < 0.001, Fig. 2b) suggested the best cut-off point was 245 U/L with a specificity of 69.4% and a sensitivity of 85.9%. The ROC curve of D-dimer (AUC = 0.775, 95% CI: 0.698–0.841, *P* < 0.001, Fig. 2c) suggested the best cut-off point was 0.96 μg/mL with 77.0% specificity and 78.1% sensitivity. The ROC curve of SAA (AUC = 0.835, 95% CI: 0.764–0.892, *P* < 0.001, Fig. 2d) indicated the best cut-off point was 100.02 mg/L with 72.2% specificity and 85.9% sensitivity. The ROC curve of albumin (AUC = 0.848, 95% CI: 0.779–0.903, *P* < 0.001, Fig. 2e) indicated the best cut-off point was 36 g/L with a specificity of 83.3% and a sensitivity of 85.9%. The ROC curve of CRP (AUC = 0.776, 95% CI: 0.698–0.841, *P* < 0.001, Fig. 2f) suggested the best cut-off point was 64.79 mg/L with a specificity of 81.9% and a sensitivity of 64.8%.

**Fig. 3:**
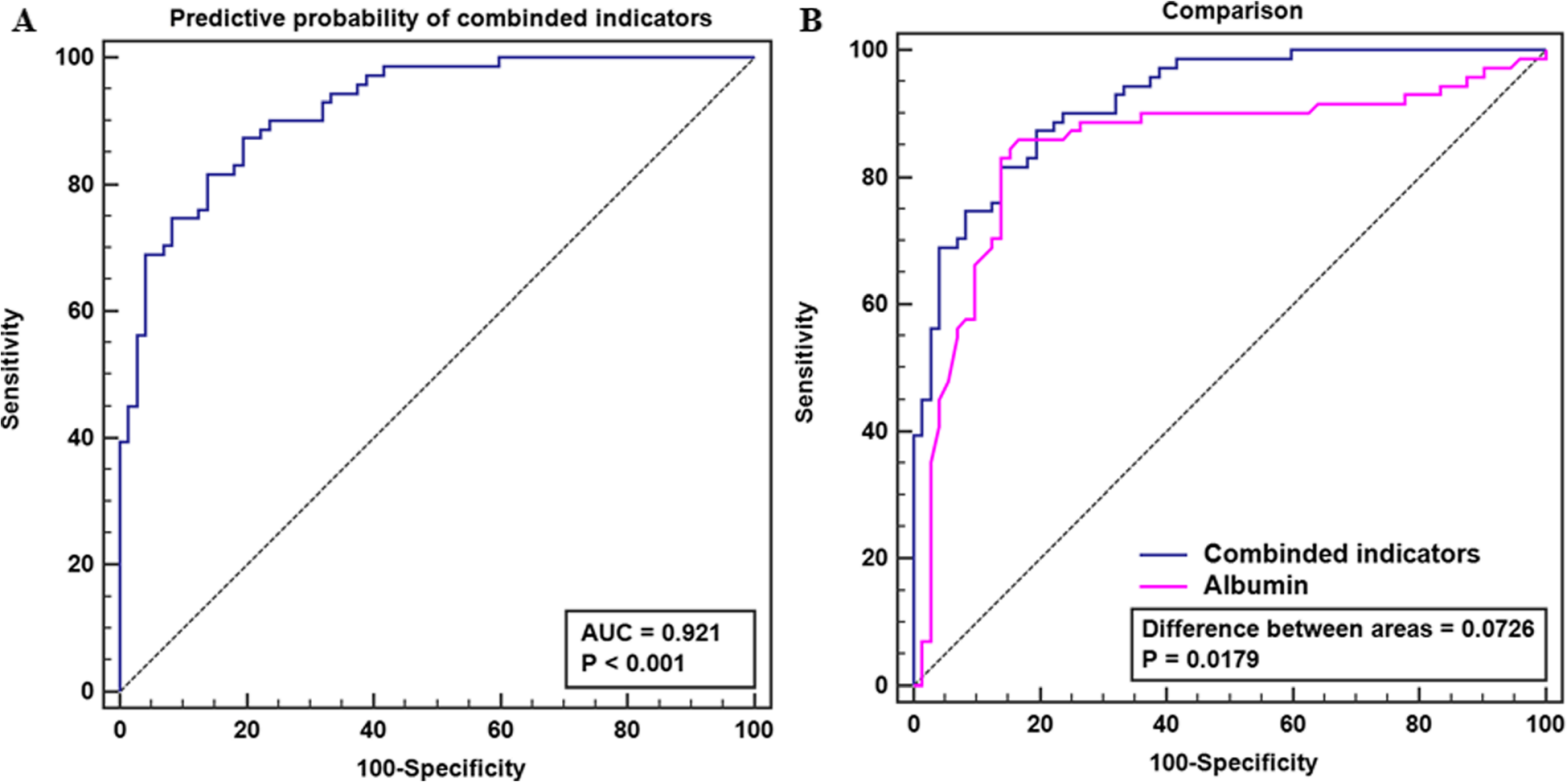
ROC curve of combined indicators for the speculation of disease severity

The area under the ROC curve for albumin was the biggest (AUC = 0.848), and the area under the ROC curve for SAA ranked second (AUC = 0.835). The calculated difference between the AUC for albumin and the AUC for SAA was 0.013, but it was not statistically significant (*P* = 0.787). Binary logistic regression was applied to calculate the predictive probability of combined indicators for the speculation of disease severity. The combined indicators found that the AUC reached 0.921 (95% CI: 0.864–0.959, *P* < 0.001, Fig. 3a), with a sensitivity of 87.3% and a specificity of 80.6%. The area difference between the combined indicators and albumin was 0.0726 (95% CI: 0.0125–0.133, *P* = 0.0179, Fig. 3b), indicating that the accuracy of the combined identification of the six indicators was the best.

## Discussion

The SARS-CoV-2 outbreaking at the end of 2019 is highly contagious and more than 80,000 patients have been reported in Chinese mainland [9]. The number of confirmed cases worldwide has risen by 7499 to 132,758, among which nearly 28,900 were reported in the European region [10]. Most patients are mild to moderate severity, and with a better prognosis [11]. However, for patients developing into severe or critical levels, the mortality rate was markedly higher. It is important to identify critically ill patients even earlier, aiming to reduce mortality and improve the recovery rate.

In this study, the relationship between disease severity and clinical and biochemical indicators was comprehensively analyzed. Most critical ill patients were older and had a greater number of comorbid conditions than patients with mild to moderate illness. This was consistent with a study [7] in which prospectively included 138 patients with COVID-19 and the researcher suggested that age and comorbidity may be risk factors for poor outcome. Moreover, our study showed that the ROC curve of age was administrated, and the best cut-off point of age was 52 years.

In accordance with Liu J’s [12] and Wan S’s [13] study, this study also found that the levels of lymphocyte count, neutrophil count and CRP were associated with the severity of COVID-19. In addition, ESR, PCT, D-dimer and SAA were also related to the disease severity. PCT levels in serum increase when severe bacterial, fungal and systemic inflammatory response syndrome occur, and it is generally not elevated with virus infections [14]. In our study, the PCT concentration in severe/critical patients was significantly higher than that in the mild/moderate group when PCT ≥ 0.04 ng/mL. It suggested the possibility of multiple infections in critically ill patients.

Besides, we also found that the levels of HDL-C and serum potassium were negatively correlated with the severity of COVID-19. According to Dong C’s [15] study, hypokalemia was prevailing in patients with COVID-19, and the correction of hypokalemia was challenging because of continuous renal K+ loss resulting from the degradation of ACE2. HDL-C was known to play protective role in a variety of disease states, including viral pneumonia [16]. Serum HDL-C levels might decrease and serum total cholesterol/HDL-C ratios might increase proportionally in community-acquired pneumonia [17]. According to Wei C’s [18] study, the spike protein of SARS-CoV-2 bound to HDL and antagonists of HDL receptor-Scavenger receptor class B type I inhibited SARS-CoV-2 infection. The lipids transfer function of Scavenger receptor class B type I was indispensable for this inhibition, providing explanations for the reduced serum HDL level in patients with COVID-19. In a retrospective study [19] reporting on 97 adults with laboratory-confirmed COVID-19, Decrease in HDL-C was found to be valuable in predicting the transition of COVID-19 from mild to severe illness. Similar results were obtained in our own research. Therefore, serum potassium and HDL-C levels may be a good prognostic index.

In this study, there were also some other abnormal indicators that had significant differences between the mild/moderate and the severe/critical group, such as plasma glucose, TBIL, AST, ALT, and LDH. These abnormalities suggested that SARS-CoV-2 infection may be associated with myocardia injury, hepatic injury and other related organ damage. Based on the ROC analysis between mild/moderate and severe/critical patients, some cut-off values of the test items were obtained. With age > 52 years, CRP > 64.79 mg/L, LDH > 245 U/L, D-dimer > 0.96 μg/mL, SAA > 100.02 mg/L, albumin < 36 g/L, progress to critical illness should be closely observed and prevented.

According to Lu J’s study [5], a simple mortality risk index, composed of age and CRP, can predict COVID-19 related short-term mortality. In a retrospective cohort study [20] reporting on 191 adult inpatients with laboratory-confirmed COVID-19, multivariable regression showed increasing odds of in-hospital death associated with older age (odds ratio 1.10, 95% CI 1.03–1.17, per year increase; *p* = 0.0043) and D-dimer greater than 1 μg/mL (18.42, 2.64–128.55; *p* = 0.0033) on admission. According to Wu C’s study [21], for the patients with COVID-19, risk factors associated with the development of ARDS and progression from ARDS to death included older age (hazard ratio [HR], 3.26; 95% CI 2.08–5.11; and HR, 6.17; 95% CI, 3.26–11.67, respectively), higher LDH (HR, 1.61; 95% CI, 1.44–1.79; and HR, 1.30; 95% CI, 1.11–1.52, respectively) and D-dimer (HR, 1.03; 95% CI, 1.01–1.04; and HR, 1.02; 95% CI, 1.01–1.04, respectively). So far, no study has shown plasma glucose, TBIL, AST and ALT were independent risk factors for COVID-19 progression. It is possible that these may be confounding factors, which still needs to be further confirmed.

According to the above, patients’ conditions on admission including old age, comorbidities, lymphocytopenia, hypoalbuminemia, and other abnomal indicators may predict the severity of the disease. These factors need further investigation and should be considered for risk stratification. We have found that COVID-19 progressed rapidly for some critically ill patients. Therefore, for those at high risk, close monitoring and timely treatment might be very important and could help to improve the outcome.

This study has several limitations. First, it is a pity that some inflammatory factors and immunological indexes cannot be detected and compared due to the limitation of experimental conditions. Second, this is a cross-sectional study and participants were from 1 center rather than multiple centers. It provides no information regarding cause or effect relationship. Although we found significant associations, further studies are needed to investigate clinical significance of these indicators on patients with COVID-19.

## Conclusions

With following parameters such as age > 52 years, C-reactive protein > 64.79 mg/L, lactate dehydrogenase > 245 U/L, D-dimer > 0.96 μg/mL, serum amyloid A > 100.02 mg/L, or albumin < 36 g/L, the progress of COVID-19 to critical stage should be closely observed and possibly prevented. Lymphocyte count, serum potassium, high-density lipoprotein cholesterol and procalcitonin may also be a prognostic indicator.

## Data Availability

All data generated or analyzed during this study are included in this published article. The datasets used and/or analysed during the current study are available from the corresponding author on reasonable request.

## Abbreviations

COVID-19: Coronavirus disease 2019
SARS-CoV-2: Severe acute respiratory syndrome coronavirus 2
WHO: World Health Organization
ARDS: Acute respiratory distress syndrome
ICU: Intensive care unit
ESR: Erythrocyte sedimentation rate
CRP: C-reactive protein
PCT: Procalcitonin
IQR: Interquartile range
ROC: Receiver–operating characteristic
HDL-C: High-density lipoprotein cholesterol
LDL-C: Low-density lipoprotein cholesterol
TBIL: Total bilirubin
AST: Aspartate aminotransferase
ALT: Alanine aminotransferase
LDH: Lactate dehydrogenase
SAA: Serum amyloid A
BUN: Blood urea nitrogen
CK-MB: Creatine kinase-muscle and brain type
APTT: Activated partial thromboplastin time
PT: Prothrombin time
AUC: Area under the ROC curve
HR: Hazard ratio
